# Classifying the Binding Modes of Disordered Proteins

**DOI:** 10.3390/ijms21228615

**Published:** 2020-11-16

**Authors:** Monika Fuxreiter

**Affiliations:** 1 Department of Biomedical Sciences, University of Padova, 35131 Padova, Italy; monika.fuxreiter@unipd.it; 2 Laboratory of Protein Dynamics, University of Debrecen, 4032 Debrecen, Hungary

**Keywords:** protein interaction, disordered protein, fuzzy binding, context-dependence, conformational heterogeneity

## Abstract

Disordered proteins often act as interaction hubs in cellular pathways, via the specific recognition of a distinguished set of partners. While disordered regions can adopt a well-defined conformation upon binding, the coupled folding to binding model does not explain how interaction versatility is achieved. Here, I present a classification scheme for the binding modes of disordered protein regions, based on their conformational heterogeneity in the bound state. Binding modes are defined as (i) **disorder-to-order transitions** leading to a well-defined bound state, (ii) **disordered binding** leading to a disordered bound state and (iii) **fuzzy binding** when the degree of disorder in the bound state may vary with the partner or cellular conditions. Fuzzy binding includes polymorphic bound structures, conditional folding and dynamic binding. This classification scheme describes the structural continuum of complexes involving disordered regions as well as their context-dependent interaction behaviors.

## 1. Introduction

Interactions mediated by disordered protein regions are critical for the regulation of numerous cellular pathways [1,2]. Although the benefits of structural plasticity in interaction versatility have been recognized [3], the molecular mechanisms and biophysical principles have remained rather enigmatic. The key questions are (i) the origin of selectivity, (ii) affinity versus specificity, (iii) selectivity for multiple partners and (iv) the plasticity of the interactions depending on the cellular conditions. Here, I aim to approach these problems by providing a classification system for the different binding modes of disordered regions.

The specific binding of disordered regions is generally interpreted via a coupled folding to binding model [4], when conformational ensembles of disordered regions adopt a well-defined structure upon interacting with the target protein [5]. The structure may differ between complexes with different partners, including changes in the secondary structure. The binding-competent conformation can be achieved via ‘conformational selection’, when it is transiently sampled in the free protein, or via an ‘induced fit’, when a conformation that is not sampled in the free state is realised in the bound structure [6]. Although preformed secondary structures are frequently observed in complexes involving disordered regions [7], increasing experimental evidence underscores the plasticity of the templated folding pathway [8]. In particular, the highly heterogeneous conformational ensemble in the transition state of folding is often controlled by interactions outside the binding interface (e.g., in the c-Myb:KIX interaction [9]). Non-native interactions also appear to modulate affinity and contribute to selectivity [10].

Extensive fluctuations of the well-defined secondary structure elements in the bound state of disordered proteins underscore the complexity of the templated folding pathway [11]. Dynamics at the binding interface enables to sample alternative contacts, which may serve as a basis of selectivity for different partners [12]. Indeed, the multivalency or redundancy of binding motifs can result in highly dynamical, heterogeneous bound states [13]. Furthermore, increasing experimental evidence shows that disordered regions may remain disordered in their bound states [14]. Biomolecular condensates, for example, formed by liquid-liquid phase separation demonstrate that specific interactions can be achieved without a well-defined bound-state structure [15]. Thus, it is increasingly recognised that the bound state of disordered regions samples a continuum between order and disorder, which cannot be captured by a coupled folding to binding model.

The plasticity of the bound state also enables changes in interactions depending on the partner, cellular localisation or posttranslational modifications. Systematic studies show that most disordered regions sample both ordered and disordered states in their complexes [16]. Phosphorylation may, for example, induce a transition between ordered and disordered states in the bound complex, which may trigger further binding events or polymerisation [17,18]. The fine-tuned equilibrium between ordered and disordered states in the complexes of disordered proteins often contributes to their regulatory roles. All these observations prompt a model that can describe the large variety and plasticity of bound conformations, along with their sensitivity to the cellular environment.

Here, I present a classification scheme for interactions of disordered regions. This approach is based on a biophysical framework related to conformational entropy, which can also be applied to predict binding modes from sequence.

## 2. Results

### 2.1. The Framework for Defining Binding Modes

**Figure 1:**
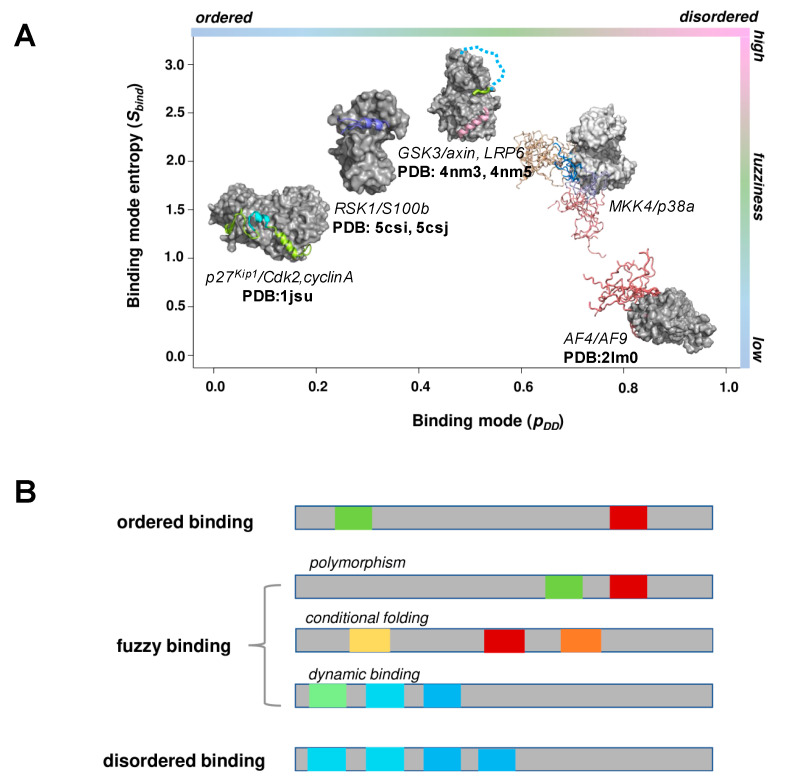
**Binding modes of disordered regions.** (**A**) **Binding mode landscapes of representative complexes.** The *x*-axis represents the probability of disordered binding (p_DD_), and the *y*-axis represents the binding mode entropy (*S_bind_*), the Shannon entropy, of possible binding modes. Both quantities were computed using the FuzPred program [16,44], and the structures represent the p_DD_ and *S_bind_* of the disordered binding region averaged over the residue-values. **The disorder-to-order binding mode** is exemplified by p27^Kip1^, which folds into an α-helical conformation upon binding to Cdk2/cyclinA (PDB:1jsu) [20]. Some parts of the helix, which are also sampled in the unbound state, adopt a stable structure in the complex (residues 51–63, *cyan*), while the helical conformation of other regions may vary with different partners [51] (*lime*). **Polymorphic binding** is exemplified by ribosomal S6 kinase 1, which interacts with S100β via different secondary structures (PDB: 5csn, 5csi, 5csj, 5csf) [22]. **Conditional folding** is represented by the N-terminal region of glycogen-synthase 3, which folds upon phosphorylation in the insulin pathway, while it remains disordered in the Wnt pathway (PDB: 4nm3, 4nm5) [23]. **Dynamic binding** is exemplified by MAPK kinase 4 upon binding to p38a, which establishes a dynamic interaction profile involving regions outside the canonical motif (coordinates as a courtesy of Malene Ringkjobing–Jensen) [24]. **Disordered binding** is represented by AF4 binding to leukemia fusion target AF9 (PDB:2lm0), where affinity-modulating residues remain to be disordered in the complex [26]. (**B**) **Sequence patterns of the interacting motifs in different binding modes.** Disorder-to-order transitions involved a few, well-defined motifs (*red, green*). Disordered binding is achieved via multiple, weak-affinity motifs (*blue*). Fuzzy binding is usually achieved via multiple motifs, from which one can be distinguished under given conditions. The distinction between the motifs decreases towards more dynamic binding modes.

The bound state of disordered regions can vary along a continuum between order and disorder. ‘Binding modes’ are defined by the probability of disorder in the bound state (p_DD_) [16]. This characterizes the conformational heterogeneity in the assembly and can be related to the change in conformational entropy upon binding. One of the extremes of the scale (p_DD_ = 0) is an ordered bound state, which refers to a well-defined, unique conformation (Figure 1A). This state excludes alternative structures or alternative contact patterns resulting from conformational variations or dynamics in the assembly as well as conditional folding, which occurs only with given partner(s) or posttranslational modifications. The other extreme of the scale (p_DD_ = 1) is the disordered bound state, which is defined by many different binding configurations, including a variety of conformations and contact patterns (Figure 1A). Thus, the disordered bound state is composed of many different microstates and has a high conformational entropy. In between these two extremes, a continuum of bound states represents different degrees of disorder or conformational heterogeneity. Disordered regions with intermediate p_DD_ values frequently sample different binding modes (Figure 1A). First, the two extremes of the binding mode continuum will be discussed, followed by an analysis of the fuzzy binding modes (Table 1).

### 2.2. Disorder-to-Order Transition

Disordered proteins often exhibit secondary structure elements, which resemble their binding competent conformation [27]. Interactions of such preformed recognition elements with the target shifts the ensemble towards the bound-like conformation, which will be dominantly present in the resulting assembly. In this manner, interactions with the partner considerably reduce the number of conformations available in the unbound state. Binding to a partner may also stabilise a conformation, which is rarely sampled in the free-state ensemble (‘induced fit’) [28]. Both the conformational selection and induced fit result in well-defined, ordered bound states, termed as disorder-to-order binding modes (Figure 1A).

Defining an ordered bound state may appear straightforward, but in practice it is rather complicated. The templated folding of disordered regions dramatically differs from a normal folding process [29]. Sequences of disordered regions contain short, low-specificity interaction motifs, which form non-native contacts [30,31]. The presence of non-native interactions leads to multiple, often competing folding pathways, which may result in different secondary structures in the bound form [32]. Non-native interactions may induce fluctuations of the structured binding elements, leading to different positions/arrangements at a shallow binding interface [33,34]. The heterogeneous transition state of templated folding might enable selectivity for multiple partners [8].

*Disorder-to-order binding modes are defined by a single, well-defined, fully ordered binding configuration.* Disorder-to-order binding modes exclude binding-coupled transitions with variations in the bound conformation, including changes in secondary structure, conformational parameters (e.g., torsion angles) or the position of the bound structure. The disorder-to-order binding mode is mediated by a unique, well-defined contact pattern, excluding ambiguities.

Disorder-to-order binding modes are low-entropy states, which are driven by distinguished binding motifs.

### 2.3. Disordered Binding

Upon interactions, disordered regions can also retain their conformational heterogeneity in the bound state. For example, the interaction between the highly charged prothymosin-α with histone H1, an ultrahigh affinity complex, is achieved without a considerable ordering of the binding site [25]. In this case, the contacts can be realised between any pairs of charged residues, leading to many possible binding configurations. Along the same lines, Sic1 binds to the same of region Cdc4 via nine redundant phosphorylation sites [35]. The bound complex is established by alternative contacts between any of the phosphorylation sites and the target, with a dynamic exchange between these bound configurations.

*Disordered binding modes are defined by many different binding configurations.* These assemblies are represented by an ensemble of conformations with comparable energies and not by a unique structure in the bound state (Figure 1A). Consequently, disordered binding modes are realised by alternative contact patterns, often amongst weak or redundant motifs [36]. The most pertinent examples are proteins undergoing a liquid-liquid phase separation [37]. Although specific sequence motifs may be identified, the linking regions also contribute considerably to the binding process via the mediation of the exchange between the different binding configurations.

The biophysical principles of disordered binding, in particular regarding the origin of specificity, have not been fully understood [38]. Disordered binding is generated by energetically comparable native and non-native interactions, or between redundant native contacts, which both result in a rugged energy landscape. This reflects the fact that even in the presence of a partner these sequences are not compatible with folding. The specificity of disordered binding is likely encoded in the distinguished physico-chemical properties of the binding residues as compared to their flanking sequences [16]. Thus, the interactions between the individual motifs are weak, but the avidity of the interaction can be large [39].

Disordered binding modes are high-entropy states, which are driven by a set of weak binding motifs.

### 2.4. Fuzzy Binding

Conformational ensembles of disordered regions can be modulated in the bound state, leading to different interaction behaviours. In these cases, the protein region encodes a pool of binding modes, with different degrees of conformational heterogeneity in the bound state. The actual binding mode that is realised in the complex depends on the partner or cellular conditions. Therefore, the binding mode is context-dependent, as it is influenced by external factors (Figure 1A). This is in contrast to disorder-to-order and disordered binding modes, where the bound state is not dependent on the environment.

Fuzzy binding modes are defined by alternative binding configurations, which change with the cellular conditions. Fuzzy binding modes include polymorphism, when the protein region adopts different ordered conformations with different partners; conditional folding, when the disordered region adopts a labile structural element, which is formed under specific circumstances; and dynamic binding, when intermolecular contacts are occasionally sampled (Figure 1A).

*Polymorphism* is exemplified by disordered regions of amyloids, which self-assemble and fold into tightly packed zippers [40]. The β-strands can interact in different registers, resulting in different bound conformations and enabling different prion strains and phenotypes [41,42]. Similarly, polymorphism may lead to the activation/inhibition of alternative signaling pathways [22]. *Conditional folding* is exemplified by disordered regions, which may fold upon binding or remain disordered in the bound complex depending on the partner or posttranslational modifications. For example, phosphorylation of the eukaryotic translation initiation factor 4E-BP2 at T37 and T46 induces a labile structure at the 18–62 region, which reduces the binding affinity for eIF4E [43]. This example also corroborates the fact that structure formation may not be a prerequisite for high-affinity interactions. Conditional folding can also be induced in a complex with a specific partner. The kinase activation loop of the dual activity enzyme Ire1 remains disordered in the bound state but can fold upon autophosphorylation, which induces further oligomerisation to increase the enzymatic activity [17]. Dynamic binding is exemplified by disordered regions, which exhibit a conformational exchange in the bound complex while establishing transient contacts with the partner. The MAP kinase MKK4, for example, forms a dynamic signaling complex with p38α via sampling both free-like and bound-like states while in contact with the partner [24]. Dynamic interactions involve both the canonical binding motif and the KIS domain, which together finetune the signaling specificity. The dynamic conformational ensembles in the bound state can be modulated by the cellular conditions or other partners, which can shift the disordered region towards a more ordered or disordered state.

Fuzzy binding covers a wide spectrum of bound states between order and disorder ranging from a disorder-to-order transition to disordered binding [44] (Figure 1A). Indeed, most disordered regions sample both ordered and disordered states when bound with specific partners [16]. Fuzzy binding is generated by competing native and non-native contacts, which generate a rugged energy landscape [45]. In contrast to disordered binding, however, in this scenario the native interaction can be stabilised under certain conditions (specific target, a posttranslational modification, pH, ionic strength, concentration of given metabolites). In this manner, fuzzy binding can be manifested as disordered binding under some conditions and as more ordered binding under other conditions. The plasticity of the conformational ensemble in the bound state thus results in a wide range of interaction behaviours that result in versatile functional outcomes.

Fuzzy binding modes are high-entropy states driven by a multiple binding motifs, which under given circumstances can occasionally sample a low-entropy state.

### 2.5. Sequence Codes for Binding Modes of Disordered Proteins

Versatile interaction behaviours of disordered regions make it difficult, in particular, to identify a distinguished structure or sequence motifs, which drive binding towards selected partners. While some secondary structures may be helpful, stabilising their conformations may compromise affinity [46]. Early observations have, together with later systematic studies [47], demonstrated the importance of non-native interactions in shaping the characteristics of the final complex [48].

Thus, to predict the binding mode of a disordered protein region, one needs to search for potential interaction sites that may compromise the binding motif (if any). This reverts the original question from chasing a pre-structured element to the analysis of competing interactions. Solving this problem requires a completely different computational approach. To decide whether a disordered region undergoes a disorder-to-order transition upon binding or remains disordered in the bound state, one needs to analyze similarities between any potential binding site and its environment (i.e., flanking sequence). In case the motif is unique and distinguished from its local sequence space, it will drive the disordered region towards a well-defined bound state (Figure 1B). If the motif is redundant or exhibits similarities to the neighboring sequences, structural ordering will be compromised. This scenario results in disordered binding modes (Figure 1B). These principles serve as a basis of the FuzPred method, which predicts the binding modes of disordered regions based on local sequence biases [16]. The method gives a high performance prediction on the continuum of binding modes of disordered regions, and was validated on ~2000 protein complexes [16,44]. In addition, disordered binding modes were used to predict protein liquid-liquid phase separation, and the results were validated by experiments [49].

Obviously, the potential binding motifs are further influenced by extrinsic factors, which also affect their competition. Posttranslational modifications, the presence of specific interaction partners, the ionic strength of the cellular milieu and a wide variety of factors modify the characteristics of the potential binding motifs. This reshapes the interaction energy landscape, leading to fuzzy binding, polymorphism, conditional folding or dynamic binding (Figure 1A). Fuzzy binding reflects the functional variability of disordered regions, which is challenging to quantify. Following the FuzPred approach, one can determine the local sequence biases using different sequence environments representing different, hypothetical binding sites [44]. This provides a distribution of probabilities for disorder-to-order [50] or disorder-to-disorder transitions {p_DD_} upon interactions with many potential partners. The Shannon entropy of this distribution defines the ‘entropy’ of binding modes (*S_bind_*), whose computational protocol has been previously published [44]. The ‘entropy’ of binding modes informs one to what extent the binding mode is well-defined and to what extent it will vary with the cellular conditions. Fuzzy binding modes have high *S_bind_* values, while regions exhibiting a uniform binding mode in all their known complexes (disorder-to-order or disorder-to-disorder) have low *S_bind_* values (Figure 1A). The computational protocols on how to assign the different modes have been published previously [16,44].

Taken together, these recent computational developments demonstrate that the wide range of interaction behaviours of disordered regions are encoded in sequence and can be predicted without information on the specific binding partner.

## 3. Concluding Remarks

It has been increasingly recognized that complexes of disordered regions span the continuum between order and disorder, depending on the interplay between a variety of short interaction motifs [3]. We are just beginning to realise that interaction behaviours can also change, depending on the partner, posttranslational modifications or cellular environment. The proposed classification scheme provides a quantitative framework to describe these interaction behaviours based on conformational entropy. In accordance with this, we define the interaction behaviours based on (i) the change of disorder upon binding (p_DD_) and (ii) the dependence of the binding mode on the environment (S_bind_). This scheme results in a landscape of binding modes defined by the p_DD_ and S_bind_ values. The arbitrary division of the landscape results in five distinct interaction behaviours, which can be predicted from sequence. Quantitative definitions for the different binding modes open perspectives for modulating protein interactions that regulate complex cellular processes.

## Figures and Tables

**Table 1:** Classification of the binding modes of disordered regions.

Binding Mode	Bound Conformation	Contact Pattern	Interactions	Example
**Disorder-to-order**	ordered	well-defined	permanent	p53 oligomerisation domain [19], p27^Kip1^ [20]
**Fuzzy binding**, *polymorphic*	ordered	multiple	permanent	Sup35 [21], RSK1 [22]
**Fuzzy binding**, *conditional folding*	ordered or disordered	multiple	permanent/transient	Ire1 [17], GSK3 [23]
**Fuzzy binding**, *dynamic*	disordered	multiple	transient	MKK4 [24]
**Disorder-to-disorder**	disordered	multiple	transient	Prothymosine [25], AF4 [26]
